# Enhancing flexibility mindset: a randomized controlled trial using daily emotion-focused self-reflection

**DOI:** 10.3389/fpsyg.2026.1965838

**Published:** 2026-10-12

**Authors:** Dirk W. Eilert, Lola-Gia Schaper, Frauke Meyer, Anne Weigand, Philipp Mensah, Austin Barkhorn, George A. Bonanno

**Affiliations:** 1 Institute of Psychology, University of Innsbruck, Innsbruck, Austria; 2 Eilert Institute for Emotion Science, Berlin, Germany; 3 Institute of Neuroscience and Biopsychology for Clinical Application, MSB Medical School Berlin, Berlin, Germany; 4 Department of Clinical and Counseling Psychology, Teachers College, Columbia University, New York, NY, United States

**Keywords:** coping, emotion processing, emotion regulation, mindset, regulatory flexibility

## Abstract

**Introduction:**

Flexibility mindset has recently been proposed as a motivational factor that supports adaptive self-regulation during adversity, yet it remains unknown whether it can be strengthened through psychological intervention. The present randomized controlled trial examined whether a brief daily emotion-focused self-reflection intervention designed to cultivate six emotional resources (authentic pride, awe, flow, gratitude, relaxation, and vicarious joy) would increase flexibility mindset and improve mental health outcomes.

**Methods:**

Forty-four university students were randomly assigned to either an intervention group or an active control group and were instructed to complete daily micro-exercises over a three-week period. Assessments were conducted at baseline, post-intervention, and a two-week follow-up and included measures of flexibility mindset, depressive symptoms, anxiety, stress, positive affect, and negative affect.

**Results:**

Participants in the intervention group showed significant increases in flexibility mindset from pre to post (within-group *d* = 0.91) and from pre to follow-up (within-group *d* = 1.05), whereas no significant changes were observed in the active control group. Between-group contrasts confirmed greater increases in the intervention group at both assessments. Across both conditions, higher flexibility mindset was associated with higher positive affect at post and follow-up after adjustment for baseline flexibility mindset, baseline positive affect, and group, with both associations remaining significant after Holm correction. Depressive symptoms, anxiety, and stress decreased significantly over time across conditions, whereas no significant change in negative affect was detected.

**Conclusion:**

To our knowledge, this is the first randomized controlled study to provide evidence that flexibility mindset may be amenable to psychological intervention. The findings suggest that the deliberate cultivation of emotional resources may represent a promising pathway for strengthening motivational processes relevant to adaptive self-regulation. Further research is needed to examine underlying mechanisms, long-term effects, and generalizability across populations and contexts.

## Graphical abstract

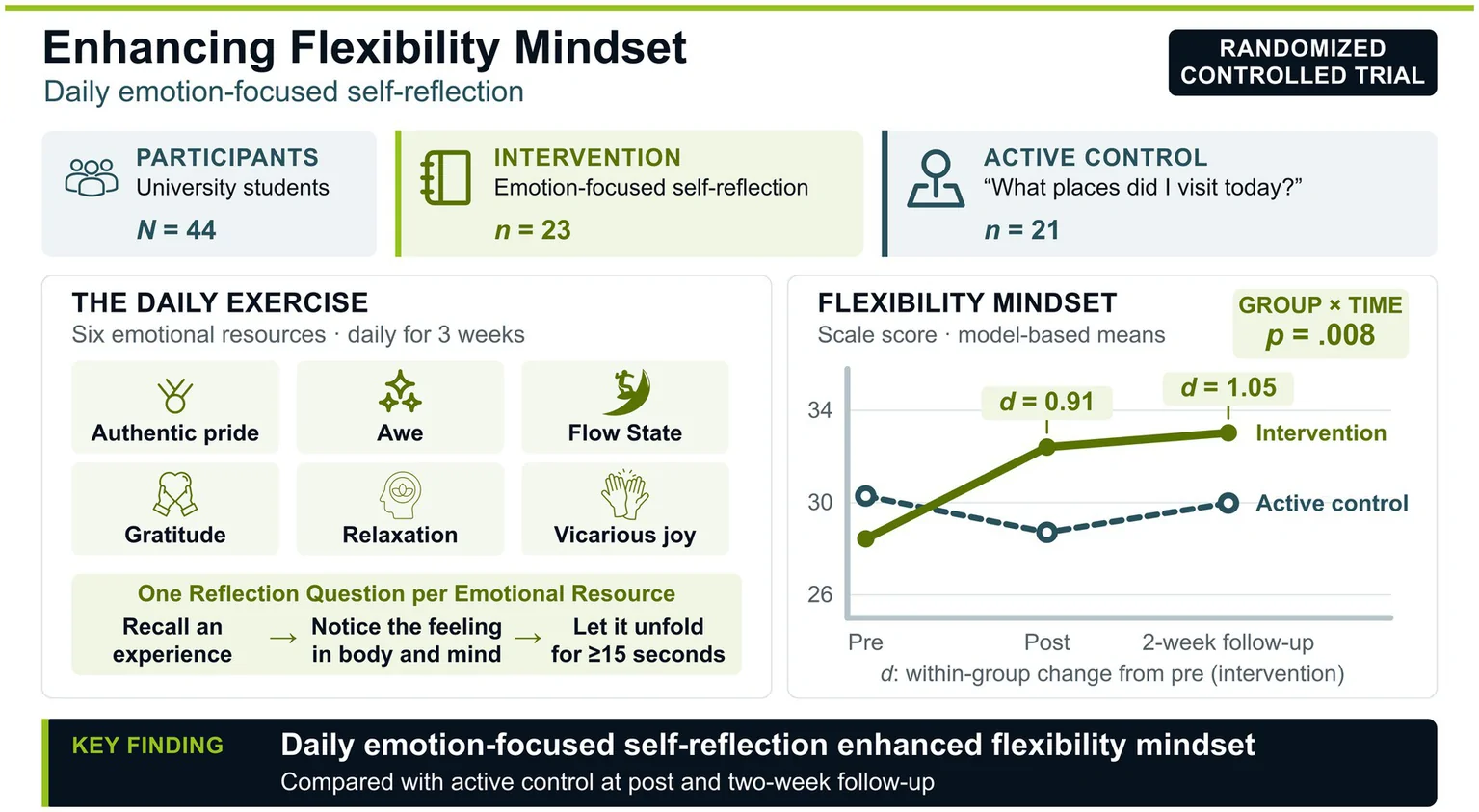

## 1 Introduction

Most people will encounter highly stressful, potentially traumatic, or otherwise adverse life events at some point in their lives. Serious illness, bereavement, interpersonal loss, accidents, natural disasters, and other major stressors are not exceptional experiences but part of the human condition. Given the ubiquity of such events, understanding why some individuals maintain psychological health whereas others experience persistent distress has become a central question in resilience research.

Across a wide range of potentially traumatic and highly adverse life events, resilience has consistently emerged as the most common outcome trajectory. Longitudinal research has shown that most individuals maintain relatively stable psychological functioning following adversity, whereas only a minority develop persistent psychological impairment (Galatzer-Levy et al., 2018; Bonanno et al., 2023). This pattern has been observed across diverse stressors, including serious illness, natural disasters, bereavement, interpersonal loss, and potentially traumatic events. These findings challenge traditional assumptions that psychological distress is the normative response to adversity and instead suggest that humans possess a remarkable capacity for adaptation.

At the same time, identifying the factors that reliably predict resilient outcomes has proven surprisingly difficult. Although numerous variables, including optimism, coping strategies, social support, self-efficacy, and pleasant emotions, have been associated with resilience, their individual predictive effects are often modest and highly dependent on contextual factors (Bonanno, 2021; Bonanno et al., 2023). This observation has been described as the resilience paradox: resilience is common, yet no single protective factor appears sufficient to explain why some individuals adapt successfully across a broad range of challenging circumstances. Consequently, recent resilience research has increasingly shifted its focus away from isolated traits and toward adaptive self-regulatory processes.

One influential framework that has emerged from this process-oriented perspective is *regulatory flexibility*. Regulatory flexibility describes adaptive self-regulation as a dynamic process through which individuals respond to changing situational demands rather than relying on fixed coping styles or universally effective strategies (Bonanno and Burton, 2013; Bonanno et al., 2023). According to this framework, successful adaptation depends on the ability to accurately evaluate contextual demands, access a diverse repertoire of potential responses, and continuously monitor whether a chosen response is producing the desired outcome. These three components – context sensitivity, repertoire, and feedback monitoring – together form what has been termed the *flexibility sequence*.

A growing body of research suggests that the effectiveness of any regulatory strategy depends less on the strategy itself than on its fit with situational demands. Strategies that are adaptive in one context may be ineffective or even counterproductive in another (Bonanno and Burton, 2013; Aldao et al., 2015). Consequently, resilience is not viewed as the result of consistently applying a particular coping strategy but rather as the capacity to flexibly adjust one’s responses as circumstances change. This perspective helps explain why resilience has proven difficult to predict from isolated traits or behaviors and highlights the importance of adaptive regulatory processes (Bonanno and Westphal, 2024).

At the same time, possessing regulatory skills does not necessarily guarantee that individuals will engage in flexible self-regulation when facing adversity. Effective adaptation often requires sustained effort, tolerance of uncertainty, and repeated adjustments in response to setbacks (Bonanno et al., 2023). Particularly under conditions of stress, individuals may disengage from challenges, perceive demands as overwhelming, or doubt their ability to cope effectively. To account for this motivational dimension, recent work has introduced the concept of *flexibility mindset* (Bonanno, 2021).

Flexibility mindset has been conceptualized as the optimistic conviction that one can and will do what is necessary to get through a difficult situation. Rather than representing a specific coping strategy, it reflects a motivational orientation that supports engagement with the flexibility sequence. Within the broader regulatory flexibility framework, flexibility mindset represents the motivational component, whereas the flexibility sequence describes the mechanistic processes of adapting using the serially related components of context sensitivity, repertoire, and feedback monitoring. The present study assessed this motivational component rather than these regulatory processes.

Conceptually, flexibility mindset is theorized to arise from a set of mutually reinforcing beliefs, including optimism, coping self-efficacy, and challenge appraisal (Bonanno, 2021; Barkhorn et al., 2025). Optimism promotes the expectation that favorable outcomes remain possible despite current difficulties. Coping self-efficacy reflects confidence in one’s ability to manage aversive situations effectively. Challenge appraisals are evaluations of demanding situations not solely as threats but also as opportunities for learning, growth, or successful adaptation (Jamieson et al., 2018).

Together, these beliefs may increase the likelihood that individuals actively engage in adaptive self-regulation and persist in the face of adversity. Recent evidence supports this proposition. Barkhorn et al. (2025) found that optimism and coping self-efficacy strengthened the association between regulatory flexibility and mental health outcomes, suggesting that motivational factors may enhance the effectiveness of flexible self-regulation.

These findings raise an important question: if flexibility mindset contributes to adaptive functioning, can it be deliberately strengthened through brief, scalable interventions that can be integrated into everyday life?

One promising pathway for strengthening flexibility mindset may lie in the deliberate cultivation of emotional resources. Although emotions are often conceptualized as consequences of successful adaptation, an increasing body of research suggests that emotional experiences can also function as antecedents of adaptive functioning. In particular, pleasant emotional experiences appear to play an important role in building psychological resources that support coping and resilience over time.

This perspective is reflected in Fredrickson’s broaden-and-build theory, which proposes that pleasant emotions temporarily expand individuals’ thought-action repertoires, fostering greater openness, cognitive flexibility, exploration, and the recognition of novel possibilities for action (Fredrickson, 2004). Repeated experiences of pleasant emotions are thought to accumulate into enduring psychological, social, and behavioral resources that can support adaptive functioning during future challenges. Consistent with this proposition, prospective research following the September 11 terrorist attacks found that individuals who experienced more pleasant emotions during the aftermath of the crisis reported greater resilience and psychological growth over time (Fredrickson et al., 2003). Moreover, pleasant emotions appear to facilitate recovery from adverse emotional experiences and support adaptive adjustment in the face of stress, suggesting that they may contribute to resilience rather than merely reflect it (Tugade and Fredrickson, 2004).

Recent work has further suggested that individuals can intentionally shape their emotional experiences through deliberate practices. This process has been described as *emotion crafting* and refers to the intentional creation, amplification, and maintenance of desirable emotional states in everyday life (van der Kaap-Deeder et al., 2023). Rather than passively waiting for beneficial emotional experiences to occur, individuals may actively cultivate emotional resources that support adaptive functioning. From this perspective, brief daily self-reflection exercises aimed at strengthening specific emotional resources may represent a scalable and accessible approach for fostering the motivational foundations of adaptive self-regulation.

Building on these considerations, the present intervention focused on the deliberate cultivation of six emotional resources through a brief daily self-reflection exercise. The intervention was designed to strengthen emotional experiences that have been linked to adaptive functioning and psychological well-being while simultaneously promoting a balanced emotional resource profile.

Three of these emotional resources were selected because of their conceptual links to core components of flexibility mindset. *Authentic pride* was included because it reflects successful goal pursuit and has been associated with perseverance, achievement motivation, and sustained engagement in challenging tasks (Williams and DeSteno, 2008; Weidman et al., 2016; Gilchrist et al., 2018). These characteristics closely resemble the confidence in one’s ability to manage adversity that underlies coping self-efficacy. *Awe* was included because it broadens attention, promotes perspective taking, and reduces self-focused rumination (Rudd et al., 2012; Stancato and Keltner, 2021; van Elk et al., 2019). Experimental research further suggests that awe can foster optimism, reduce self-doubt, and increase confidence when facing future challenges (Le et al., 2019; Pan and Jiang, 2024), making it theoretically relevant to the optimism component of flexibility mindset. *Flow* was included because it emerges when individuals engage with challenges that stretch but do not exceed their perceived abilities (Moneta and Csikszentmihalyi, 1996). By fostering engagement with demanding yet manageable situations, flow may reinforce the tendency to perceive challenges as opportunities for growth and mastery rather than as threats. Consistent with this notion, flow has been associated with more adaptive appraisals of demanding situations and greater psychological functioning under challenge (Mao et al., 2020).

Importantly, the intervention was not designed solely to strengthen these motivational beliefs. Consistent with regulatory flexibility theory, adaptive functioning is unlikely to depend on maximizing any single psychological resource. Rather, effective adaptation requires a flexible balance of complementary emotional and motivational resources that can be recruited according to situational demands (Kashdan and Rottenberg, 2010; Bonanno and Burton, 2013). Excessive confidence, unchecked striving, or overly optimistic expectations may become maladaptive when they are insufficiently balanced by acceptance, grounding, and social connectedness. For example, unrealistically optimistic beliefs can lead to inaccurate risk perceptions and reduced adaptive preparation (Shepperd et al., 2013), whereas high self-efficacy may become counterproductive when individuals persist in attempting to control fundamentally uncontrollable situations (Schiaffino et al., 1991). More broadly, this perspective is consistent with emerging concepts of *metastability*, which emphasize the capacity to flexibly transition between multiple adaptive states rather than remaining fixed in any single mode of responding (Hellyer et al., 2015; Kotler et al., 2026).

Therefore, gratitude, relaxation, and vicarious joy were incorporated as complementary emotional resources. *Gratitude* has been associated with acceptance, positive reappraisal, and reduced self-focused striving (Bartlett and DeSteno, 2006; Lambert et al., 2009; Wood et al., 2009; DeSteno et al., 2014). *Relaxation* promotes physiological and psychological downregulation and may counteract chronic activation and perseverative thinking (Sakakibara et al., 1994; Teixeira et al., 2005; Gangadharan and Madani, 2018). *Vicarious joy*, defined as sharing in the happiness and successes of others, has been associated with greater social connectedness, interpersonal warmth, and prosocial orientation (Zeng et al., 2017a; Zeng et al., 2017b; Major et al., 2018). By directing attention toward positive experiences in the lives of others, vicarious joy may foster a greater sense of connection and appreciation within social relationships.

Together, these six emotional resources of authentic pride, awe, flow, gratitude, relaxation, and vicarious joy may help maintain a flexible balance between engagement and acceptance, striving and grounding, and individual agency and social connectedness.

Taken together, these considerations suggest that the deliberate cultivation of these six emotional resources may represent a promising pathway for strengthening flexibility mindset. By repeatedly activating emotional experiences linked to optimism, coping self-efficacy, challenge appraisal, acceptance, grounding, and social connectedness, such an intervention may support the motivational foundations of adaptive self-regulation. At the same time, the cultivation of these emotional resources may contribute to broader improvements in psychological well-being through their effects on positive affect and resilient adaptation.

The present study examined whether a brief daily emotion-focused self-reflection intervention designed to cultivate six emotional resources associated with adaptive self-regulation and flexibility mindset would strengthen flexibility mindset and improve mental health outcomes compared with an active control condition. Based on the theoretical framework outlined above, we hypothesized that participants in the intervention group would show greater increases in flexibility mindset over time than participants in the active control group (H1). Furthermore, we expected a higher flexibility mindset to be associated with higher levels of positive affect at post-intervention and follow-up (H2). Finally, across both conditions, participants were expected to show reductions in depressive symptoms, anxiety, stress, and negative affect over time (H3).

## 2 Materials and methods

### 2.1 Sample description and acquisition

The sample consists of German university students in their early and mid-adulthood. Eligible participants were individuals aged 18 to 35. Participants were recruited via social media, the SONA system, and personal referrals. The exclusion criterion was a self-reported neurological or psychiatric disorder. Participation in the study was voluntary. Students at the MSB Medical School Berlin received research participant credits through the SONA system for their participation; no further compensation was offered.

After completing the baseline assessment (T0), all 44 participants were randomly assigned to the intervention group (IG; *n* = 23) or the active control group (ACG; *n* = 21) using automated randomization in SoSci Survey (SoSci Survey GmbH, Munich, Germany) with the default balanced-allocation setting. The procedure was set up by a member of the research team. The research team neither knew participants’ assignments in advance nor intervened in the allocation process. Participants were not told whether they were in the intervention or active control group.

Before the start of the study, all participants received detailed information about the study and provided informed consent. The study was approved by the Institutional Ethics Committee of the MSB Medical School Berlin, Berlin, Germany (approval no. MSB-2025/320; approved on 17 November 2025), and was conducted in accordance with the Declaration of Helsinki.

An *a priori* power analysis was conducted with G*Power (Version 3.1.9.6; Heinrich-Heine-University Düsseldorf, Düsseldorf, Germany; Faul et al., 2007). Because G*Power does not directly support power calculations for linear mixed-effects models, a repeated-measures ANOVA testing the group × time interaction was used as an approximation, with two groups and three measurement occasions. Assuming a medium effect size of *f* = 0.25, α = 0.05, a target power of 0.95, a correlation of *r* = 0.50 between repeated measurements, and a nonsphericity correction of ε = 1, the required total sample size was *N* = 44. This was the recruitment target; no additional allowance for dropout was included. IG consisted of *n* = 23 participants at each of the measurement time points T0 and T1, and *n* = 22 at T2. ACG consisted of *n* = 21 participants; at T2, *n* = 20. One participant in each group did not complete the follow-up assessment (T2); no reason for dropout was reported. Participant flow from randomization through follow-up and analysis is shown in Figure 1.

**Figure 1:**
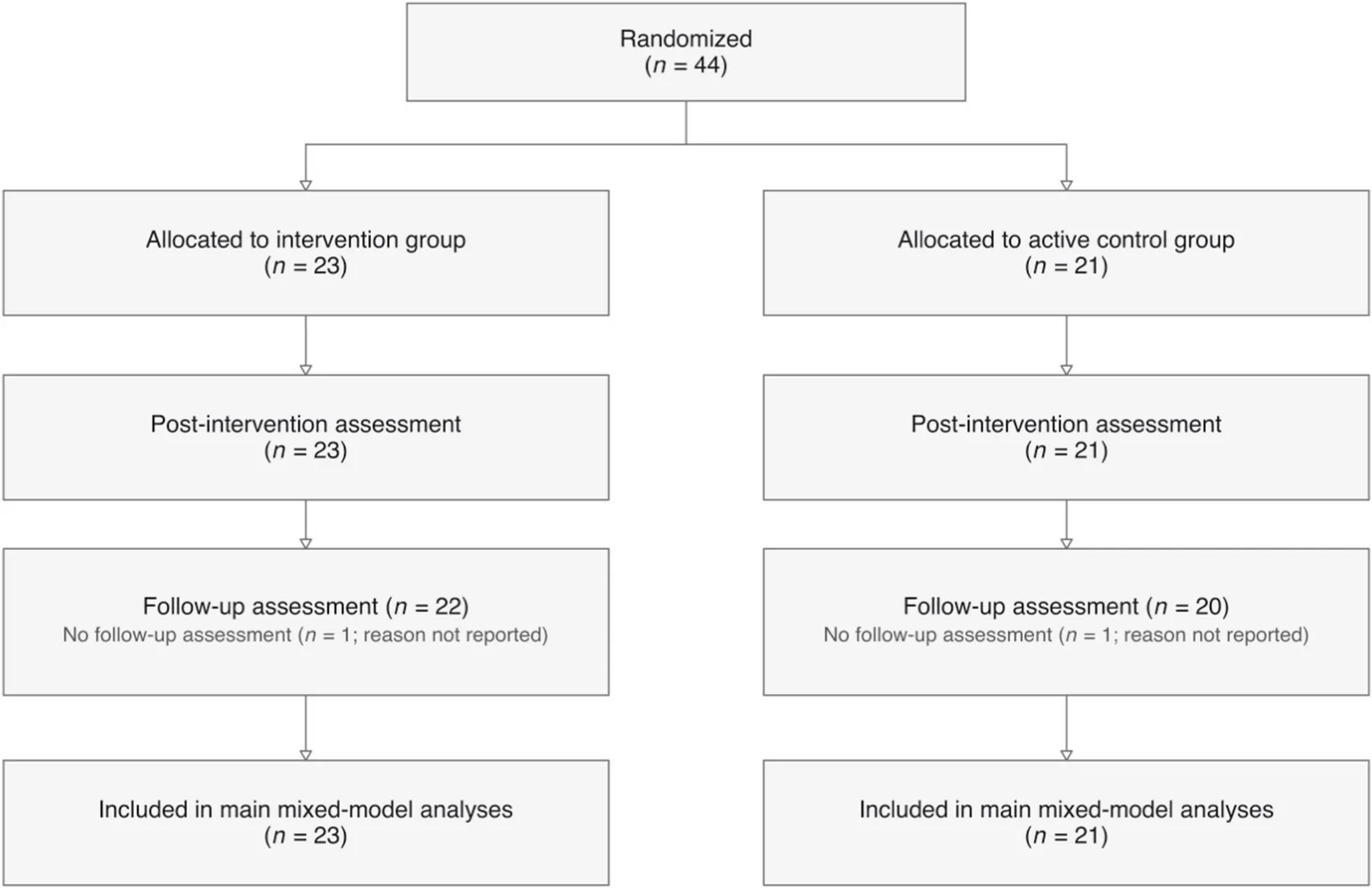
Participant flow through allocation, post-intervention assessment, follow-up, and the main mixed-model analyses. Models used available observations, including those from participants without follow-up. One intervention-group participant had a missing FMS score at post-intervention; this participant’s baseline and follow-up FMS observations were retained.

Participants’ gender, age, study degree, and current semester were measured as personal variables. IG (*M* = 24.5 years, *SD* = 4.34 years) did not differ significantly in age from ACG (*M* = 26.1 years, *SD* = 3.86 years), *t*(42) = −1.30, *p* = 0.20. No significant differences were observed in gender composition (IG: 78.3% female; ACG: 66.7% female), (Fisher’s exact test, *p* = 0.49), study degree (Fisher’s exact test, *p* = 0.37), and current semester (Fisher’s exact test, *p* = 0.29).

### 2.2 Study design and procedure

The experiment was conducted as a randomized controlled longitudinal study using a pre-post-follow-up design. Participants were randomly assigned to one of two treatment conditions: IG or ACG. Both groups were exposed to daily self-reflection micro-exercises for a period of three weeks.

Dependent variables were assessed at three time points: T0 (pre), prior to the start of the micro-exercises; T1 (post), immediately after completing the three-week intervention; T2 (follow-up), two weeks after the intervention.

Flexibility mindset was the focal outcome of the intervention hypothesis (H1). Positive affect was examined in the association analyses addressing H2, whereas depressive symptoms, anxiety, stress, and negative affect were examined as additional outcomes addressing H3. The trial, hypotheses, outcome hierarchy, and statistical analysis plan were not prospectively registered. The distinction between the focal and additional outcomes reflects the organization of the research questions and does not represent a prospectively documented primary–secondary outcome hierarchy; the analyses therefore provide hypothesis-based exploratory evidence.

Data collection was conducted in a single concurrent cohort between November 2025 and January 2026, with all participants completing the three assessments according to the same schedule.

The following section details the measures used to assess these outcomes at each time point.

#### 2.2.1 Measures

The *Flexibility Mindset Scale (FMS)* assesses flexibility mindset, defined as the optimistic conviction that one can and will do what is necessary to get through a difficult situation (Barkhorn and Bonanno, 2026, manuscript submitted for publication). The scale comprises six items reflecting both positively and negatively framed responses to challenging situations and has demonstrated a unidimensional structure, good internal consistency (Cronbach’s alpha ranging from 0.82 to 0.88), and convergent validity with related constructs such as optimism and coping self-efficacy (Barkhorn and Bonanno, 2026, manuscript submitted for publication). The event-specific version of the scale (FMS-E), which was used in the present study, has been shown to better differentiate the construct from trait-level measures and to capture both stable individual differences and situational variability in responses to stress.

The FMS-E was translated from English into German by a native German speaker with English as a foreign language. An English back-translation was generated using ChatGPT (ChatGPT-5.5, OpenAI, San Francisco, CA, USA), without providing the original English items in the back-translation task. The back-translated items were subsequently reviewed against the originals in consultation with one of the scale developers. No substantive conceptual discrepancies were identified during this review. No separate pilot testing or cultural adaptation study was conducted before data collection. The German translation had not undergone independent psychometric validation before its use in the present study.

At each assessment, participants were instructed to think of an extremely difficult event from their own lives that was distressing, aversive, or potentially traumatic and to provide a brief label that would allow them to remember it. We did not verify whether they referred to the same event across measurement occasions; therefore, the event selected may have differed between assessments. Using the stem “Faced with this event, I tended to …,” participants rated the extent to which each of the six items described their response to the event on a 7-point Likert scale ranging from 1 (“does not apply to me at all”) to 7 (“applies to me completely”), with higher scores indicating a stronger flexibility mindset. Example items include “Vertrauen in meine Fähigkeit zu spüren, mit der Situation umzugehen” (“Feeling confident in my ability to deal with the situation”) and “Mich besiegt zu fühlen” (“Feeling defeated”). Internal consistency of the German FMS-E in the present sample was α = 0.85 at T0, α = 0.77 at T1, and α = 0.81 at T2.

*Depressive symptoms, anxiety, and stress* were assessed using the respective subscales of the German version of the Depression Anxiety Stress Scales (DASS-21; Nilges and Essau, 2015). The DASS-21 comprises 21 items measuring three dimensions of psychological distress: depression (D), anxiety (A), and stress (S). Participants rated the extent to which each statement applied to them over the past week on a 4-point Likert scale ranging from (0) “did not apply to me at all” to (3) “applied to me very strongly or most of the time.” Example items include “Ich war nicht in der Lage, mich für irgendetwas zu begeistern” (“I was unable to become enthusiastic about anything”) for depression, “Ich spürte, dass mein Mund trocken war” (“I was aware of dryness of my mouth”) for anxiety, and “Ich fand es schwer, mich zu beruhigen” (“I found it hard to wind down”) for stress.

The German version of the DASS-21 demonstrates good internal consistency, with Cronbach’s alpha ranging from 0.78 to 0.92 across subscales (Nilges and Essau, 2015). These findings are supported by additional evidence from Bibi et al. (2020), reporting alpha values between 0.81 and 0.90. Convergent validity has been demonstrated through significant correlations with established measures such as the Beck Depression Inventory (BDI) and the Beck Anxiety Inventory (BAI), with coefficients ranging from 0.56 to 0.76 (Nilges and Essau, 2015). For the stress subscale, Bibi et al. (2020) reported negative correlations with positive mental health (*r* = −0.61), life satisfaction (*r* = −0.43), social support (*r* = −0.33), and resilience (*r* = −0.41) in their German sample, supporting its construct validity.

*Positive affect* and *negative affect* were assessed using the German version of the Positive and Negative Affect Schedule (PANAS; Breyer and Bluemke, 2016). The PANAS comprises 20 items measuring positive affect (PA) and negative affect (NA). Participants rated the extent to which they experienced each affective state on a 5-point Likert scale ranging from (1) “not at all” to (5) “extremely.” Example items include “aktiv” (“active”) and “interessiert” (“interested”) for positive affect, and “schuldig” (“guilty”) and “bekümmert” (“distressed”) for negative affect.

The German version of the PANAS demonstrates good internal consistency, with Cronbach’s alpha values around 0.86 for both subscales (Breyer and Bluemke, 2016), with comparable results reported in other studies (Leue and Beauducel, 2011). Convergent and criterion validity have been supported through associations with related constructs such as life satisfaction, happiness, extraversion, neuroticism, and health indicators (Leue and Beauducel, 2011; Breyer and Bluemke, 2016).

#### 2.2.2 Interventions

Participants in IG were instructed to complete a daily micro-exercise designed to cultivate six emotional resources over a period of 3 weeks. The exercise aimed to deliberately evoke emotional experiences associated with adaptive self-regulation and flexibility mindset in everyday life. Participants in this group were instructed to complete six emotion-focused self-reflection questions each day, targeting authentic pride, awe, flow, gratitude, relaxation, and vicarious joy. The six emotion-focused self-reflection questions were taken directly from Eilert (2021) and selected on the theoretical grounds outlined in the Introduction to target the respective emotional resources (see Table 1). Participants were instructed to take a moment for each question, imagine a situation in which they had experienced the corresponding emotional resource, and notice how the experience felt in their bodies. In doing so, they were asked to attend to bodily sensations as well as mental and emotional aspects, and to consciously allow the feeling to unfold for at least 15 s. Participants were instructed to repeat this procedure for each of the six emotional resources. Based on prior practical experience, the daily exercise was estimated to take approximately 5 minutes; actual completion times were not recorded.

**Table 1:** Micro-exercise: emotion-focused self-reflective questions.

Emotional resource	Short description	Emotion-focused self-reflective question
Authentic Pride	A feeling when we achieve something through our own actions that is personally meaningful to us.	“Was habe ich heute durch mein Handeln erreicht, auf das ich stolz bin?” (“What did I achieve today through my actions that I feel proud of?”)
Awe	An uplifting sense of wonder that arises when we encounter something greater than ourselves—such as the beauty of nature, a special moment, or a person who deeply moves us.	“Wo ist mir heute ein ‘Wunder’ begegnet, wann habe ich Ehrfurcht gespürt?” (“When did I experience a sense of awe today, or encounter something that felt like a ‘wonder’?”)
Flow State	A state of deep focus and effortless engagement in which we are fully absorbed in an activity and lose awareness of time and surroundings.	“In welchem Moment bin ich heute in einer Tätigkeit voll aufgegangen und habe alles um mich herum vergessen? Wann habe ich ein Flow-Gefühl empfunden?” (“At what moment today was I fully absorbed in an activity and lost awareness of everything around me? When did I experience a state of flow?”)
Gratitude	A warm feeling that arises when we consciously notice the good things we encounter in life—through other people, circumstances, or life itself.	“Wofür bin ich heute dankbar?” (“What am I grateful for today?”)
Relaxation	A state of inner calm in which the body and nervous system can recover and regulate.	“In welchem Moment habe ich heute Entspannung gefühlt?” (“When did I feel relaxed today?”)
Vicarious Joy	A pleasant feeling that arises when we experience joy in response to another person’s happiness and feel connected to them.	“Was war heute ein Moment der Mitfreude, in dem ich mich mit einem anderen Menschen bzw. einem anderen Lebewesen positiv verbunden gefühlt habe?” (“What was a moment today in which I felt joy for someone else and experienced a sense of positive connection?”)

During the same period, participants in ACG were instructed to answer a daily self-reflection question with non-emotional content: “What places did I visit today?” This active control condition was designed to control for nonspecific effects of daily self-reflection, including self-monitoring, reflection on daily experiences, cognitive self-distancing, and the structuring of daily experiences (Lyubomirsky and Layous, 2013; Kross and Ayduk, 2017). However, the conditions were not matched for the number of prompts or task demands, limiting the extent to which group differences can be attributed specifically to the emotion-focused content.

Daily exercise completion was not systematically monitored or recorded in either group. Consequently, adherence rates, the number of completed sessions, and deviations from the prescribed exercise schedule could not be quantified. No manipulation check was conducted to assess whether the exercises increased the six targeted emotional resources.

## 3 Results

### 3.1 Descriptive statistics

Descriptive statistics for all outcome variables across the three measurement occasions (pre, post, follow-up) are presented in Table 2. Internal consistency estimates across scales and measurement occasions ranged from Cronbach’s α = 0.73 to 0.94.

**Table 2:** Descriptive statistics of the scales at each time of measurement.

Scale (number of items)	Time of measurement	*M*	*SD*	Cronbach’s *α*
FMS (6)	Pre	29.32	6.79	0.85
	Post	30.53	5.74	0.77
	Follow-up	31.52	5.68	0.81
DASS-D (7)	Pre	4.77	4.37	0.89
	Post	4.14	3.87	0.86
	Follow-up	3.12	2.86	0.81
DASS-A (7)	Pre	5.09	4.52	0.84
	Post	4.00	3.41	0.80
	Follow-up	2.62	2.98	0.81
DASS-S (7)	Pre	7.75	4.97	0.90
	Post	6.11	3.59	0.78
	Follow-up	5.19	3.10	0.73
PANAS-PA (10)	Pre	3.18	0.65	0.87
	Post	3.15	0.81	0.94
	Follow-up	3.20	0.73	0.90
PANAS-NA (10)	Pre	1.70	0.51	0.83
	Post	1.58	0.45	0.84
	Follow-up	1.55	0.44	0.82

Baseline: *N* = 44; post: *N* = 44, except FMS (*n* = 43); follow-up: *N* = 42. Cronbach’s α values are unstandardized.

To examine baseline comparability between conditions, questionnaire outcomes at T0 were compared between IG and ACG. Welch’s *t*-tests indicated no significant pre-intervention differences between IG and ACG in flexibility mindset, depressive symptoms, anxiety, stress, positive affect, or negative affect. All *p*-values were >0.05, with the smallest *p*-value being 0.288.

### 3.2 Hypothesis testing

Linear mixed-effects models were fitted with a participant-specific random intercept to account for repeated measurements. For all six outcomes, fixed effects included group, time, and the group × time interaction to assess whether changes differed between conditions. Time was treated as categorical, with pre-intervention (baseline), post-intervention, and follow-up as its three levels. The active control group and baseline served as the reference categories. H1 was evaluated using the group × time interaction for flexibility mindset, supplemented by within-group time comparisons and between-group contrasts of change. To test the reductions over time across both conditions hypothesized in H3, additional additive models included group and time without their interaction for depressive symptoms, anxiety, stress, and negative affect. These models estimated common time effects, whereas the interaction models assessed differences in trajectories between groups. All mixed-effects models were estimated using restricted maximum likelihood, with the Kenward–Roger small-sample adjustment used for fixed-effect tests and contrasts. The main analyses did not include demographic covariates. Sensitivity analyses additionally included age as a continuous covariate and gender as a categorical covariate to assess the sensitivity of the findings to demographic adjustment. Models were fitted using lme4 and lmerTest, with car and pbkrtest supporting fixed-effect inference and emmeans used to obtain estimated marginal means and contrasts. Unless otherwise stated, estimated marginal means and contrasts were derived from the unadjusted models. Pairwise comparisons used Holm-adjusted *p*-values (Holm, 1979).

Hypothesis 2 was tested using separate linear regression models for post-intervention and follow-up, with positive affect as the dependent variable and flexibility mindset at the corresponding assessment as the predictor of interest. The main models additionally included baseline positive affect, baseline flexibility mindset, and group. Unadjusted models were also fitted for comparison. The two tests of the flexibility mindset coefficient, one at each assessment, were adjusted using the Holm procedure within each model specification. All analyses were conducted using R (version 4.5.3; R Core Team, Vienna, Austria).

All 44 randomized participants were retained in the main mixed-effects analyses and the age- and gender-adjusted sensitivity analyses, using their available observations and original group assignments. One participant in each group did not complete the follow-up assessment. In addition, one participant in the IG had a missing FMS item response at post; the FMS score for that occasion was therefore treated as missing, while the participant’s pre and follow-up scores were retained. Scale scores required complete item responses at the respective assessment; no prorating or imputation was performed. The mixed-effects analyses used an available-data approach consistent with the intention-to-treat principle of retaining participants in their randomized groups, rather than restricting the sample to participants with complete longitudinal data. Inference relied on the missing-at-random assumption conditional on the observed data included in the models. The H2 regressions used complete cases for the variables required by each model, resulting in 43 participants at post and 42 at follow-up.

For H1, the focal FMS group × time interaction was evaluated without adjustment across outcomes. As a sensitivity analysis, Holm correction was applied across the six outcome-specific group × time interaction tests. Follow-up contrasts were adjusted separately within each outcome: across the three time comparisons within each group, across the three between-group comparisons at individual measurement occasions, and across the three between-group contrasts of change. For H3, Holm correction was applied across the four omnibus time-effect tests for depressive symptoms, anxiety, stress, and negative affect. The three pairwise time comparisons were additionally adjusted within each outcome. Confidence intervals for mean differences were pointwise 95% intervals and were not adjusted for multiple testing.

Mixed-model diagnostics included inspection of residual Q–Q plots, residuals versus fitted values, and residual distributions by group and measurement occasion. All interaction and H3 additive models converged without singular fits. Residual diagnostics indicated departures from normality and differences in dispersion across some group-by-time cells, warranting caution when interpreting model-based inference. Participant-level influence was assessed by refitting these models after omitting each participant in turn. The focal FMS group × time interaction remained significant at the nominal 0.05 level in all refits, both without and with adjustment for age and gender. The Holm-adjusted conclusions for the four H3 time effects also remained unchanged. In the separate multiplicity sensitivity analysis across six outcomes, the FMS interaction remained significant in the full sample but not consistently across the leave-one-participant-out refits. Thus, the nominal H1 finding was stable to individual omissions, whereas its significance under the six-outcome Holm correction was more sensitive.

#### 3.2.1 Intervention effect on flexibility mindset (H1)

A significant time-by-group interaction emerged for flexibility mindset, *F*(2, 81.64) = 5.19, *p* = 0.008, indicating differential trajectories across conditions. The conditional main effect of time, representing changes within the active control group (ACG), was not significant, *F*(2, 81.46) = 0.75, *p* = 0.473. The conditional main effect of group, representing the between-group difference at baseline (pre-intervention), was also not significant, *F*(1, 86.94) = 1.05, *p* = 0.308.

Groupwise analyses revealed that flexibility mindset increased significantly in the intervention group (IG) from pre to post (mean increase = 3.97, 95% CI [1.36, 6.59], *p* = 0.007) and from pre to follow-up (mean increase = 4.60, 95% CI [1.99, 7.21], *p* = 0.002). The corresponding within-group effect sizes, standardized using the model residual standard deviation, were *d* = 0.91 (95% CI [0.30, 1.51]) and *d* = 1.05 (95% CI [0.44, 1.66]), respectively. No significant change was observed between post and follow-up in the IG (*p* = 0.642), and no significant changes were observed in the ACG (all *p*-values ≥ 0.746).

Interaction contrasts confirmed that the increase in flexibility mindset was greater in the IG than in the ACG from pre to post (difference in changes = 5.55, 95% CI [1.80, 9.30], *p* = 0.013) and from pre to follow-up (difference in changes = 4.90, 95% CI [1.12, 8.68], *p* = 0.023). All reported contrast *p*-values were Holm-adjusted within their respective comparison families; confidence intervals were pointwise rather than multiplicity-adjusted.

Estimated marginal means in the ACG were 30.29 at baseline (pre-intervention), 28.71 at post, and 29.98 at follow-up. Corresponding means in the IG were 28.43, 32.41, and 33.03, respectively (see Figure 2). Taken together, these findings support Hypothesis 1.

**Figure 2:**
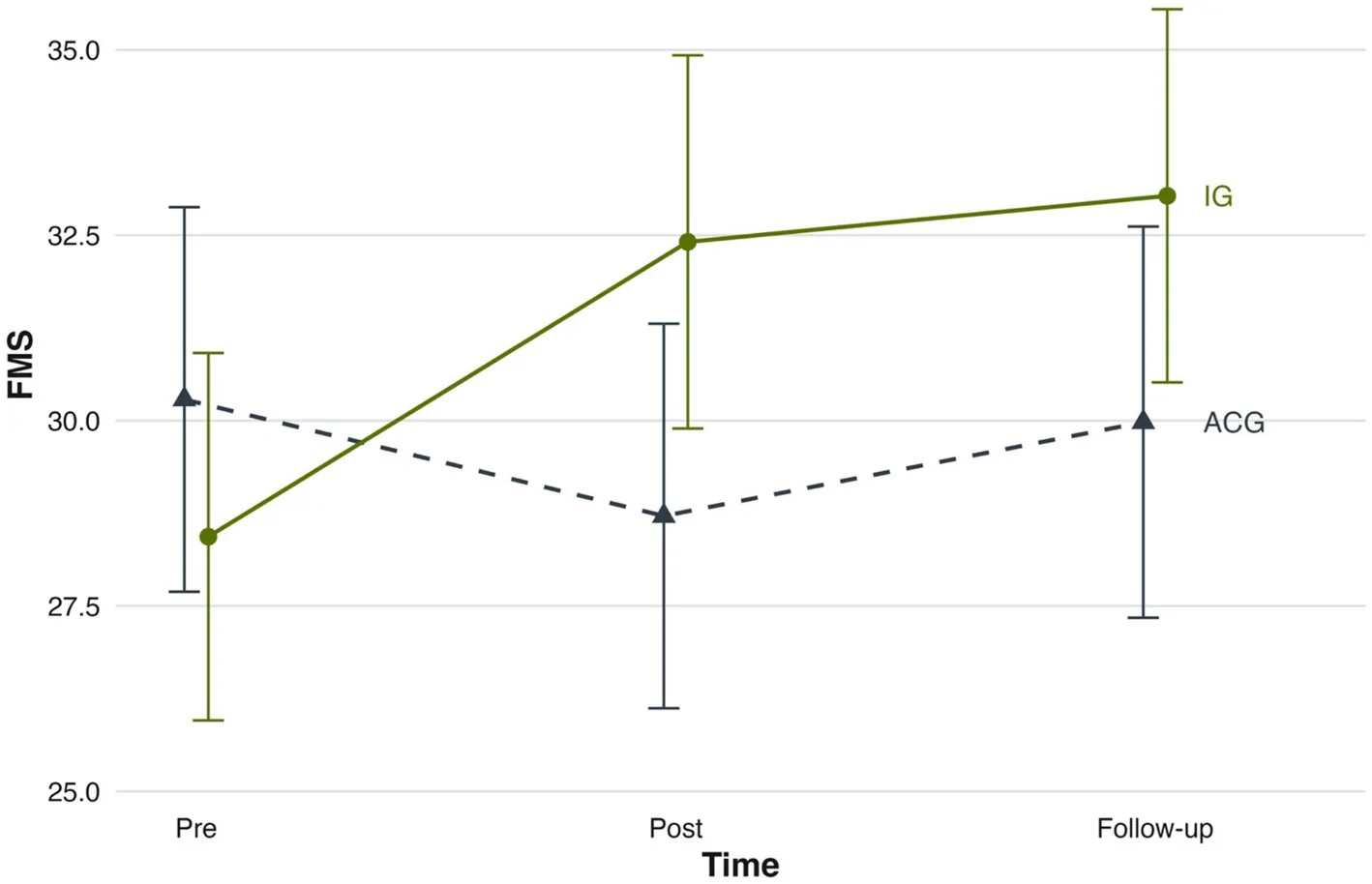
Change in flexibility mindset (flexibility mindset scale; FMS) from pre to post and follow-up by group. Values represent estimated marginal means from the linear mixed-effects model including group, time, and their interaction, with a participant-specific random intercept and without demographic covariates. Error bars indicate pointwise 95% confidence intervals. IG, intervention group; ACG, active control group.

In the age- and gender-adjusted sensitivity analysis, the time-by-group interaction remained significant, *F*(2, 81.60) = 5.19, *p* = 0.008. In the separate multiplicity sensitivity analysis across six outcomes, the interaction also remained significant in the full sample, both in the unadjusted and demographically adjusted models (both Holm-adjusted *p*-values = 0.045).

#### 3.2.2 Flexibility mindset and positive affect (H2)

Across both conditions, flexibility mindset was positively associated with positive affect at post after adjustment for baseline (pre) flexibility mindset, baseline positive affect, and group. Higher flexibility mindset was associated with higher positive affect (*b* = 0.055, *SE* = 0.023, *t*(38) = 2.40, Holm-adjusted *p* = 0.021, 95% CI [0.009, 0.101]; *n* = 43). The full model explained 22% of the variance in positive affect (*R*^2^ = 0.22, adjusted *R*^2^ = 0.13).

At follow-up, the association remained significant after adjustment for the same baseline measures and group (*b* = 0.063, *SE* = 0.021, *t*(37) = 2.97, Holm-adjusted *p* = 0.010, 95% CI [0.020, 0.106]; *n* = 42). The full model explained 36% of the variance in positive affect (*R*^2^ = 0.36, adjusted *R*^2^ = 0.29). The reported *R*^2^ values refer to the complete models, not to variance uniquely explained by flexibility mindset.

These findings support Hypothesis 2, indicating a positive contemporaneous association between flexibility mindset and positive affect at both post and follow-up beyond baseline scores and group membership. Figure 3 illustrates the unadjusted associations pooled across conditions.

**Figure 3:**
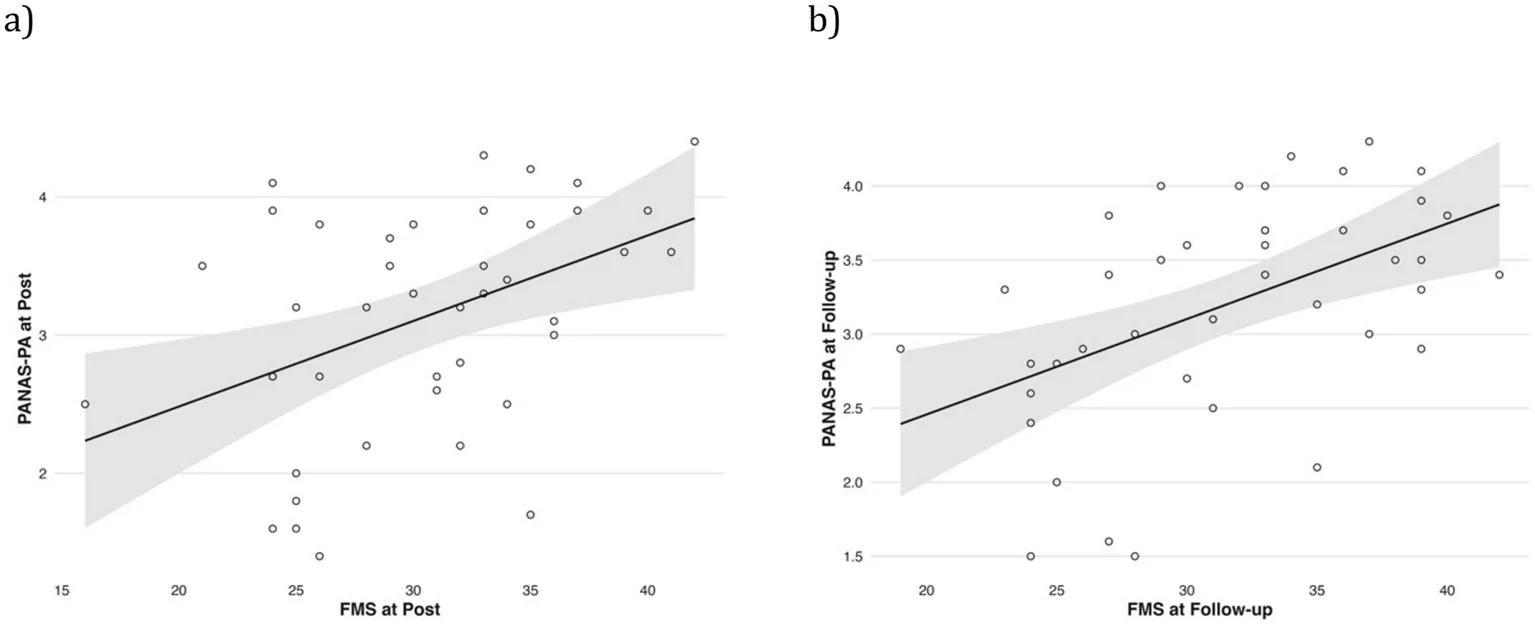
Associations between flexibility mindset (flexibility mindset scale; FMS) and positive affect (PANAS-PA) at **(a)** post and **(b)** follow-up. Each point represents an individual observation. Solid lines show unadjusted linear regression fits pooled across both groups; shaded areas indicate pointwise 95% confidence intervals for the fitted mean. These descriptive plots do not depict the baseline- and group-adjusted models reported in the main analyses.

#### 3.2.3 Changes in psychological distress and negative affect (H3)

Across both conditions, reductions over time were observed for depressive symptoms, anxiety, and stress, whereas the time effect for negative affect was not significant. The interaction models provided no evidence of differential changes between groups for these four outcomes (all nominal interaction *p*-values ≥ 0.117). Group-specific trajectories are illustrated in Figure 4. The common time effects reported below were evaluated using additive models without the time-by-group interaction. Omnibus time-effect *p*-values were Holm-adjusted across the four outcomes, and pairwise time-comparison *p*-values were Holm-adjusted across the three comparisons within each outcome. The reported *d* values standardize common time differences by the respective additive model’s residual standard deviation and do not represent between-group intervention effects. Confidence intervals are pointwise and were not adjusted for multiple testing.

**Figure 4:**
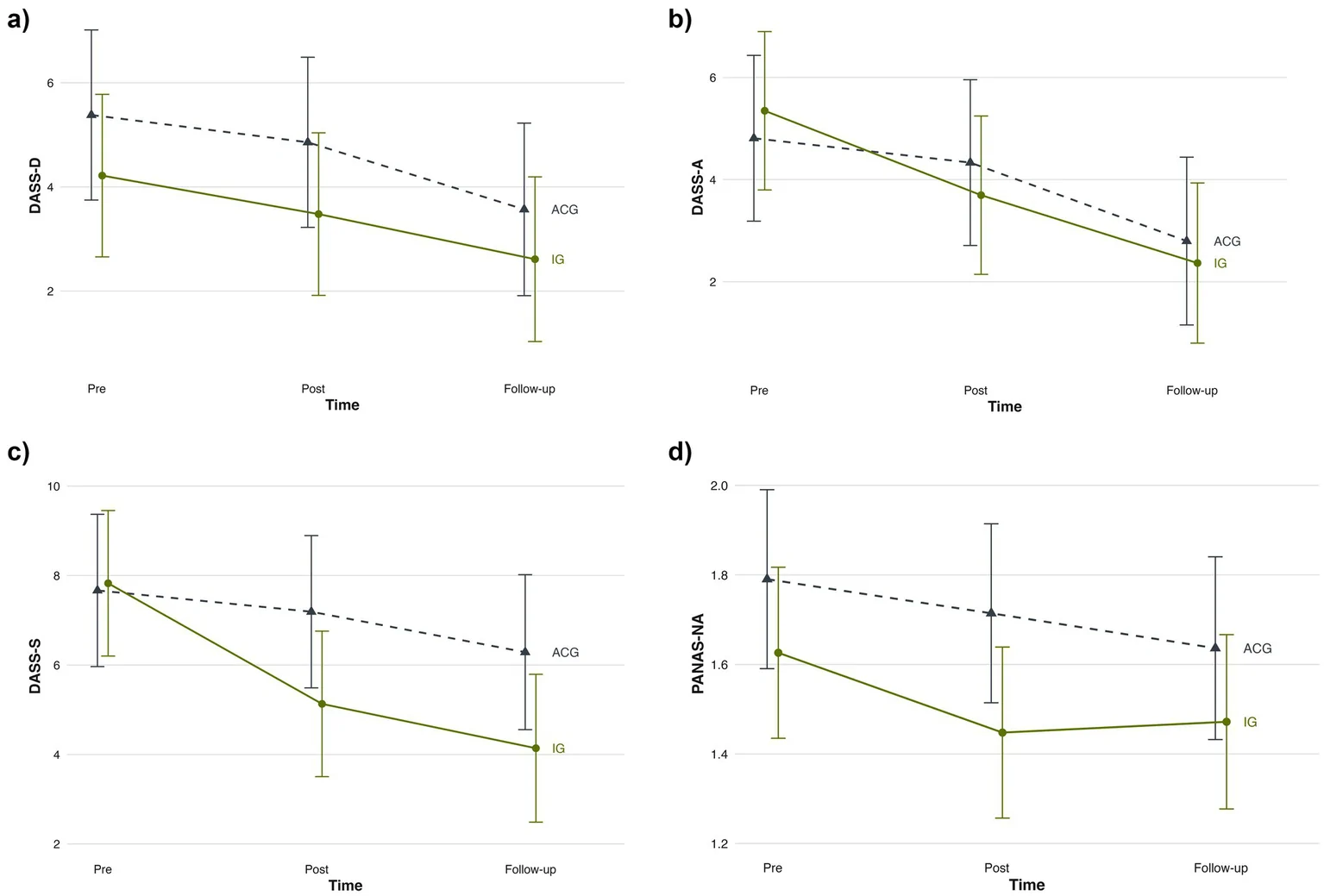
Changes in depressive symptoms (DASS-21-D) **(a)**, anxiety (DASS-21-A) **(b)**, stress (DASS-21-S) **(c)**, and negative affect (PANAS-NA) **(d)** from pre to post and follow-up by group. Values represent estimated marginal means from linear mixed-effects models including group, time, and their interaction, with a participant-specific random intercept and without demographic covariates. Error bars indicate pointwise 95% confidence intervals. The plots depict group-specific trajectories, whereas the common time effects reported for H3 were estimated using separate additive models without the interaction. IG, intervention group; ACG, active control group.

##### 3.2.3.1 Depressive symptoms (DASS-21-D)

A significant main effect of time was found, *F*(2, 84.33) = 5.28, Holm-adjusted *p* = 0.014. Pairwise comparisons indicated a significant reduction from pre to follow-up (mean decrease = 1.70, 95% CI [0.65, 2.76], *p* = 0.005, *d* = 0.70, 95% CI [0.26, 1.14]). Changes from pre to post (mean decrease = 0.64, 95% CI [−0.40, 1.67], *p* = 0.225, *d* = 0.26) and from post to follow-up (mean decrease = 1.07, 95% CI [0.02, 2.12], *p* = 0.093, *d* = 0.44) were not significant after Holm correction. The main effect of group was not significant, *F*(1, 41.98) = 1.49, *p* = 0.229.

##### 3.2.3.2 Anxiety (DASS-21-A)

A significant main effect of time emerged, *F*(2, 84.29) = 12.97, Holm-adjusted *p* < 0.001. Pairwise comparisons indicated significant reductions from pre to post (mean decrease = 1.09, 95% CI [0.12, 2.06], *p* = 0.028, *d* = 0.48), from pre to follow-up (mean decrease = 2.52, 95% CI [1.53, 3.50], *p* < 0.001, *d* = 1.10, 95% CI [0.65, 1.55]), and from post to follow-up (mean decrease = 1.43, 95% CI [0.44, 2.41], *p* = 0.010, *d* = 0.62). The main effect of group was not significant, *F*(1, 41.98) = 0.03, *p* = 0.861.

##### 3.2.3.3 Stress (DASS-21-S)

A significant main effect of time was observed, *F*(2, 84.46) = 8.49, Holm-adjusted *p* = 0.001. Pairwise comparisons indicated significant reductions from pre to post (mean decrease = 1.64, 95% CI [0.39, 2.88], *p* = 0.021, *d* = 0.56) and from pre to follow-up (mean decrease = 2.59, 95% CI [1.32, 3.85], *p* < 0.001, *d* = 0.88, 95% CI [0.43, 1.32]). The change from post to follow-up was not significant (mean decrease = 0.95, 95% CI [−0.32, 2.22], *p* = 0.140, *d* = 0.32). The main effect of group was not significant, *F*(1, 41.96) = 1.98, *p* = 0.167.

##### 3.2.3.4 Negative affect (PANAS-NA)

In contrast, the main effect of time was not significant, *F*(2, 84.60) = 2.08, Holm-adjusted *p* = 0.131. The main effect of group was also not significant, *F*(1, 41.93) = 3.71, *p* = 0.061. These results did not provide evidence of systematic changes in negative affect over time.

##### 3.2.3.5 Summary

Taken together, these findings partially support Hypothesis 3: reductions over time were observed for depressive symptoms, anxiety, and stress, but not for negative affect. These common time effects do not establish intervention-specific benefits. Although the interaction tests did not identify significant differences in trajectories between groups, this does not establish equivalence between the conditions.

## 4 Discussion

### 4.1 Interpretation of results

The present study provides the first evidence that a brief daily emotion-focused self-reflection intervention strengthens a flexibility mindset. By supporting the motivation to engage in flexible self-regulation, this intervention may ultimately contribute to more effective self-regulation and better mental health outcomes (Barkhorn et al., 2025).

Within the broader regulatory flexibility framework, the observed increases in FMS-E scores reflect stronger self-reported motivational beliefs about coping with adversity. Whether these gains also support more effective engagement with the flexibility sequence – context sensitivity, repertoire, and feedback monitoring – remains an important question for future research.

Consistent with *Hypothesis 1*, participants in the intervention group showed significant increases in flexibility mindset from pre- to post-intervention and from pre-intervention to follow-up, whereas no significant changes were observed in the active control group. Notably, these gains remained evident two weeks after the intervention had ended, suggesting that the observed changes were not limited to the immediate intervention period. The increases within the intervention group were substantial, with standardized effect sizes of *d* = 0.91 from pre to post and *d* = 1.05 from pre to follow-up. The significant time-by-group interaction and between-group contrasts of change further supported greater increases in the intervention group than in the active control group. These findings extend previous work by Barkhorn et al. (2025) linking flexibility mindset to adaptive self-regulation and mental health outcomes, suggesting that flexibility mindset itself may represent a modifiable psychological resource. More broadly, the present results are consistent with evidence indicating that adaptive self-beliefs, optimism, and self-efficacy-related cognitions can be strengthened through relatively brief psychological interventions (Toback et al., 2016; Carrillo et al., 2019; Yeager and Dweck, 2020).

*Hypothesis 2* was also supported. Across both groups, higher flexibility mindset was associated with higher levels of positive affect at both post-intervention and follow-up. Although causal conclusions cannot be drawn from these associations, the findings are consistent with theoretical accounts suggesting that adaptive motivational beliefs may facilitate engagement with challenging situations and support access to pleasant emotional experiences. More generally, the observed association aligns with research demonstrating reciprocal relationships between pleasant emotional experiences, psychological resources, and adaptive functioning (Fredrickson, 2004; Tugade and Fredrickson, 2004). These associations remained significant after adjustment for baseline flexibility mindset, baseline positive affect, and group, and after Holm correction across the two assessment occasions. Together, the predictors in the adjusted models explained 22% of the variance in positive affect at post and 36% at follow-up. Individuals who believe they can effectively cope with adversity may be more likely to remain engaged with difficult circumstances, thereby maintaining access to pleasant emotional experiences, while positive affect may in turn reinforce adaptive beliefs about coping and adjustment.

*Hypothesis 3* received partial support. Across both conditions, depressive symptoms, anxiety, and stress decreased significantly over time, whereas no significant change in negative affect was detected. Importantly, as hypothesized, these improvements were observed across both conditions rather than being specific to the intervention group. One possible explanation is that both conditions involved a structured form of daily self-reflection. Even the active control condition required participants to systematically review aspects of their daily experiences. Research on positive activity interventions suggests that engaging in regular, structured reflective activities may itself contribute to improvements in psychological well-being, even when such activities do not explicitly target emotional resources (Lyubomirsky and Layous, 2013; Davis et al., 2016). The reductions across conditions were moderate to large in standardized magnitude, with pre-to-follow-up effect sizes of *d* = 0.70 for depressive symptoms, *d* = 1.10 for anxiety, and *d* = 0.88 for stress. The absence of differential group effects suggests that these improvements cannot be uniquely attributed to the emotion-focused intervention. Although these findings are consistent with a possible benefit of structured daily self-reflection, repeated assessment, regression to the mean, and semester-related or other time-varying influences may also have contributed to the reductions in distress. The present design does not distinguish between these explanations. At the same time, the finding that flexibility mindset increased only in the intervention group suggests that strengthening motivational beliefs relevant to coping may require more than self-reflection alone and may benefit from the deliberate cultivation of specific emotional resources.

In contrast to the reductions observed for psychological distress, no significant change in negative affect was detected. This pattern may reflect important conceptual differences between psychological distress and affective experience. Whereas the DASS-21 assesses depressive symptoms, anxiety, and stress over the past week, the PANAS negative affect scale assesses the intensity of unpleasant affective states. Consequently, reductions in psychological distress do not necessarily imply corresponding decreases in the experience of unpleasant emotions. This interpretation is consistent with contemporary perspectives emphasizing that adaptive functioning depends less on the absence of unpleasant emotions than on the flexible regulation of emotional responses (Gonzalez-Escamilla et al., 2022; Bonanno et al., 2023). In addition, baseline levels of negative affect were relatively low in the present sample, which may have limited the potential for further reductions.

Taken together, the findings suggest that an intervention designed to cultivate emotional resources may be a promising approach to strengthening flexibility mindset. The reductions in psychological distress observed across both conditions are consistent with a possible contribution of structured daily self-reflection, although other time-varying influences cannot be ruled out. This pattern motivates further investigation of emotional resource cultivation as a potential mechanism underlying improvements in flexibility mindset.

### 4.2 Theoretical implications

The present findings have several implications for the emerging literature on flexibility mindset and adaptive self-regulation. First, the fact that the flexibility mindset increased only in the intervention group, whereas depressive symptoms, anxiety, and stress decreased across both conditions, suggests that changes in flexibility mindset are distinguishable from broader changes in psychological distress. This novel finding is theoretically important because it emphasizes that the flexibility mindset is not merely an individual-difference variable reflecting current mental health but rather a malleable process that can be cultivated over time. This finding aligns with contemporary perspectives emphasizing that adaptive beliefs and motivational orientations are not fixed traits but dynamic psychological resources that can be shaped through experience and intervention (Yeager and Dweck, 2020). Similar conclusions have been drawn in the literature on psychological capital (PsyCap), where meta-analytic evidence suggests that adaptive motivational resources such as optimism, self-efficacy, and hope can be strengthened through targeted intervention programs (Loghman et al., 2024).

Second, the present findings may help integrate several previously distinct lines of research. The intervention was based on the assumption that deliberately cultivating pleasant emotional experiences can strengthen psychological resources relevant to adaptation. This assumption is consistent with Fredrickson’s broaden-and-build theory, which proposes that pleasant emotions contribute to the development of enduring cognitive, psychological, and social resources that support adaptive functioning over time (Fredrickson, 2004). More recently, the concept of emotion crafting has extended this perspective by suggesting that individuals can actively cultivate desirable emotional experiences rather than merely benefiting from them when they occur spontaneously (van der Kaap-Deeder et al., 2023). The present findings are broadly consistent with these theoretical perspectives by suggesting that repeated engagement with specific emotional resources may influence motivational beliefs relevant to coping and adaptation.

At the same time, the findings complement contemporary models of resilience that emphasize adaptive self-regulation as a dynamic process. Regulatory flexibility theory proposes that successful adaptation depends on the ability to respond effectively to changing situational demands through context sensitivity, access to a diverse repertoire of responses, and feedback monitoring (Bonanno and Burton, 2013). Flexibility mindset has been conceptualized as a motivational factor that supports engagement with this flexibility sequence (Bonanno et al., 2023; Barkhorn et al., 2025). The present findings suggest that emotional resources may represent one pathway through which such motivational foundations can be strengthened. Although the underlying mechanisms remain to be investigated directly, the selective increase in flexibility mindset observed in the intervention group is consistent with the possibility that repeatedly activating emotional resources may reinforce beliefs that adversity is manageable, that effective coping is possible, and that engagement with challenges is worthwhile. This is supported by experimental evidence showing that recalling autobiographical self-efficacy episodes can promote more adaptive appraisals of negative memories and reduce distress (Paersch et al., 2022).

Finally, the present findings contribute to a growing literature suggesting that pleasant emotional experiences may function not only as consequences of successful adaptation but also as active ingredients in the development of psychological resources (Tugade and Fredrickson, 2004). While traditional resilience research has largely focused on identifying factors that predict resilient outcomes, contemporary perspectives increasingly emphasize the role of pleasant emotional processes in building capacities that support future adaptation (Fredrickson, 2004; van der Kaap-Deeder et al., 2023). This perspective is also reflected in the broader literature on positive psychology interventions. Meta-analytic evidence suggests that interventions aimed at cultivating positive cognitions and pleasant emotional experiences can enhance optimism, well-being, and related psychological resources (Carrillo et al., 2019; Carr et al., 2020).

The present findings extend this literature by suggesting that such approaches may also influence flexibility mindset. Rather than targeting coping strategies directly, the intervention focused on the repeated cultivation of emotional resources associated with adaptive functioning. The observed increase in flexibility mindset is consistent with the possibility that pleasant emotional experiences may contribute to the development of motivational beliefs that support engagement with adversity and adaptive self-regulation. Although the underlying mechanisms remain to be established, the findings support a growing view of emotions as active contributors to psychological adaptation rather than merely indicators of successful adjustment (Fredrickson, 2004; Tugade and Fredrickson, 2004). Furthermore, they suggest that the deliberate cultivation of emotional resources may represent a promising pathway for strengthening the motivational foundations of adaptive self-regulation.

### 4.3 Practical implications

The present findings may have several practical implications for interventions aimed at supporting adaptive self-regulation and psychological health. First, they suggest that interventions targeting the motivational foundations of adaptive self-regulation may be feasible. Existing approaches designed to support adaptation to stress have traditionally focused on teaching coping skills, stress-management techniques, or specific regulatory strategies. The present findings indicate that cultivating emotional resources may represent an additional pathway for supporting adaptive engagement with adversity. Rather than directly training coping behaviors, such interventions may help strengthen motivational processes that encourage individuals to remain engaged with challenges and adaptive self-regulatory efforts.

Second, the intervention evaluated in the present study was intentionally brief and easily integrated into everyday life. Participants were instructed to complete a brief daily self-reflection exercise over a three-week period. This characteristic may be particularly relevant given the growing interest in scalable, low-cost, and accessible approaches to psychological health promotion. The present findings are consistent with broader evidence suggesting that positive psychology interventions can produce meaningful improvements in well-being and related psychological resources (Carrillo et al., 2019; Carr et al., 2020). Although the observed effects require replication, the intervention format suggests a potentially practical approach for supporting emotional resources and motivational processes relevant to adaptive functioning in everyday life.

Finally, the findings suggest possible applications in preventive, educational, and coaching contexts. Universities, workplace health promotion programs, preventive mental health initiatives, and coaching settings increasingly seek brief interventions that can be implemented without extensive resources or specialist training. Interventions designed to cultivate emotional resources may offer a useful complement to existing approaches in such settings. At the same time, the present findings should be interpreted cautiously. Additional research is needed to determine whether similar effects can be observed in more diverse populations, under conditions of greater adversity, and over longer follow-up periods before broader implementation can be recommended.

### 4.4 Limitations

Several limitations should be considered when interpreting the present findings. First, the study was conducted with a relatively small sample of university students. Although significant effects emerged for flexibility mindset and effect sizes were substantial, the modest sample size limits statistical power and may reduce the stability and generalizability of the observed effects. The exploratory analyses involved multiple outcomes and comparisons. Although Holm corrections were applied within specified comparison families, they did not provide study-wide error control, and multiplicity remains relevant when interpreting the findings. Replication in larger samples is therefore needed to establish the robustness of the findings.

Second, the generalizability of the findings may be limited by our reliance on a predominantly young sample of university students from Germany. It remains unclear whether similar effects would be observed in more diverse populations differing in age, cultural background, educational level, or exposure to significant life stressors.

Third, all participants were assessed within a single concurrent student cohort from November 2025 to January 2026. Consequently, semester-related or other time-varying contextual factors cannot be disentangled from the effects of the daily self-reflection activities shared by both conditions. This limitation is particularly relevant to the decreases in depressive symptoms, anxiety, and stress observed across both groups. Future studies could address this issue by including a no-intervention control condition or by recruiting cohorts at different points in the academic year.

Fourth, the study was designed to examine changes in flexibility mindset rather than the mechanisms underlying these changes. Adherence to the daily exercises was not systematically assessed, preventing evaluation of the actual exercise dose and its relationship with outcomes. Although the intervention was designed to cultivate six emotional resources, these resources were not assessed directly. Consequently, it remains unclear whether these resources increased, whether any increases were similar across resources, whether some contributed more strongly than others, or whether changes in these resources mediated the observed increases in flexibility mindset. Future studies should therefore monitor adherence and include direct assessments of the targeted emotional resources to clarify the mechanisms through which the intervention may exert its effects.

Fifth, the active control condition provided a comparison involving daily self-reflection but was not matched to the intervention in task demands. The intervention involved six emotion-focused prompts incorporating imagery, attention to bodily sensations, and emotional processing, whereas the control condition involved a single neutral question. Differences in time spent, cognitive engagement, expectancy, perceived relevance, or task intensity may therefore have contributed to the observed group differences. The findings do not isolate the specific contribution of emotion-focused content from these other intervention characteristics. Future studies should use a control condition more closely matched on these dimensions.

Sixth, several measurement-related limitations should be considered. All outcomes were assessed by self-report and may therefore be influenced by response biases or demand characteristics. Participants were not informed whether their assigned condition was the intervention or active control condition, but the success of this masking was not assessed. In addition, although the German FMS-E showed internal consistency estimates ranging from α = 0.77 to 0.85 across assessments, the translation had not undergone independent psychometric validation. Future studies should further evaluate its psychometric properties, complement self-reports with additional assessment methods, and assess participants’ expectations and perceptions of their assigned condition.

Finally, the follow-up period was limited to 2 weeks after the intervention. Although the observed increases in flexibility mindset remained evident at follow-up, the long-term stability of these effects remains unknown. Longer follow-up periods are needed to determine whether the observed changes persist over months or whether ongoing practice is required to maintain gains over time.

Despite these limitations, the present study provides initial proof-of-concept evidence that an intervention designed to cultivate emotional resources may strengthen self-reported flexibility mindset. Adequately powered, preregistered randomized controlled trials with more closely matched control conditions and longer follow-up periods are needed to confirm and extend these findings.

### 4.5 Future research

The present findings open several avenues for future research. First, future studies investigating this emotion-focused self-reflection intervention should directly assess the emotional resources it is designed to cultivate. This would allow researchers to examine whether changes in specific emotional resources mediate the effect of the intervention on flexibility mindset. More rigorous causal tests of mediation could use experimental designs that independently manipulate both the intervention and the proposed mediator.

Second, future studies should examine whether similar effects can be achieved using alternative intervention formats. Recent advances in AI-assisted mental health interventions and conversational agents offer new opportunities to deliver brief psychological interventions in highly scalable ways. Meta-analytic evidence suggests that AI-based conversational agents can improve mental health and well-being and may represent an effective and accessible form of psychological support (He et al., 2023; Li et al., 2023). More broadly, recent reviews have highlighted the potential of AI-assisted approaches to increase accessibility, personalize interventions, and extend psychological support beyond traditional treatment settings (Olawade et al., 2024). Future research may therefore investigate whether AI-assisted interventions can effectively facilitate the cultivation of emotional resources and produce comparable changes in flexibility mindset. Such approaches may be particularly relevant for extending access to psychological support in everyday contexts while preserving the personalized and interactive elements of psychological interventions.

Third, larger studies with more diverse samples and longer follow-up periods are needed. Replication in populations differing in age, cultural background, occupational status, and exposure to adversity would help establish the generalizability of the present findings. Such research may be particularly important because evidence from positive psychology interventions suggests that the effectiveness of cultivating emotional resources can vary across cultural contexts. For example, gratitude-based interventions have been shown to produce stronger benefits in individualistic than in collectivistic cultures (Dyachenko and Trunov, 2024). Future studies should therefore examine whether the cultivation of emotional resources associated with flexibility mindset operates similarly across different cultural settings. Longer-term follow-up assessments would further clarify the stability of intervention effects and determine whether continued practice is necessary to maintain gains in flexibility mindset over time.

Finally, future research may benefit from examining flexibility mindset within the broader context of attachment theory. A growing body of research suggests that secure attachment representations are associated with more adaptive emotion regulation (Eilert and Buchheim, 2023) as well as stronger intrapersonal and interpersonal emotion perception abilities (Eilert et al., 2026). These capacities may be particularly relevant to core components of regulatory flexibility, including context sensitivity and feedback monitoring. Given emerging evidence that attachment representations and related socio-emotional capacities, including emotion regulation as well as intrapersonal and interpersonal emotion perception, may be amenable to change under certain conditions (Taylor et al., 2015; Teding van Berkhout and Malouff, 2016; Cooper et al., 2018; Sakiris and Berle, 2019; Carlucci et al., 2021), future research could investigate whether attachment-related processes contribute to the development of flexibility mindset and whether flexibility mindset may represent one pathway through which secure attachment supports adaptive functioning. Exploring these connections may help establish attachment theory as a developmental framework for understanding the emergence of flexibility mindset and adaptive self-regulation.

## Data Availability

Individual-level participant data are not publicly available due to restrictions arising from the participant information and consent procedures, which specified that study results would be reported in anonymized, group-level form. Analysis code, documentation, aggregate data, and fully synthetic example data supporting reproducibility are available at https://osf.io/s5kyq/ and are publicly accessible.
