# Supplementary material for: Enhancing flexibility mindset: a randomized controlled trial using daily emotion-focused self-reflection
Source: Front Psychol. 2026 Oct 12;17:1965838. doi: 10.3389/fpsyg.2026.1965838 (PMC13696258; doi:10.3389/fpsyg.2026.1965838)
Supplement: Supplementary file 1 [file Presentation_1.PDF]

Generative AI Prompts for Manuscript Preparation

Initial prompt:

"Please assist with the linguistic and editorial refinement of the following academic manuscript. Review the text for English grammar, spelling, punctuation, syntax, clarity, readability, coherence, flow, terminology, and academic style. Preserve the authors' intended scientific meaning, theoretical position, methodological details, statistical analyses, numerical results, effect sizes, confidence intervals, citations, interpretations, and conclusions. Do not add or invent data, methods, references, results, theoretical claims, or interpretations. Do not independently perform, modify, or reinterpret statistical analyses. Do not strengthen causal or substantive claims beyond what is supported by the study design and results. Flag passages requiring scientific judgment or clarification instead of resolving them through assumptions. Provide proposed revisions for critical review and final approval by the authors."

Final prompt:

"Please conduct a conservative final editorial review of the complete manuscript. Identify and correct only clear errors involving grammar, spelling, punctuation, syntax, readability, terminology, statistical notation, references, tables, headings, formatting, or internal consistency. Check that terminology, abbreviations, numerical results, sample sizes, statistical values, citations, tables, and statements across the abstract, main text, and discussion are consistent. Preserve the existing wording wherever no correction is necessary. Do not alter the scientific content, study design, methods, analyses, results, interpretations, conclusions, or authorial position. Do not introduce new claims, references, data, or explanations. List every proposed change so that it can be individually reviewed and approved by the authors."
